# P04.14. Acupuncture for carpal tunnel syndrome: a systematic review of randomized controlled trials

**DOI:** 10.1186/1472-6882-12-S1-P284

**Published:** 2012-06-12

**Authors:** H Shim, B Shin, M Lee, A Jung, H Lee, E Ernst

**Affiliations:** 1 Pusan National University, Yangsan, Kyngnam, Republic of Korea; 2 Korea Institute of Oriental Medicine, Daejeon, Republic of Korea; 3 Kyunghee University, Seoul, Republic of Korea; 4 Universtiy of Exeter, Exeter, United Kingdom

## Purpose

Acupuncture is a widely used symptomatic treatment for carpal tunnel syndrome (CTS). The objective of this systematic review was to evaluate the evidence of the effectiveness of acupuncture and acupuncture-like treatments for CTS.

## Methods

Systematic searches were conducted on 11 electronic databases without language restrictions. All randomized controlled trials (RCTs) of acupuncture as a treatment of CTS were included. Methodological quality was assessed using the Cochrane risk of bias tool.

## Results

Six RCTs met our inclusion criteria. Their methodological quality was generally low. Two RCTs compared the effectiveness of acupuncture with a sham control. The others used active controls. A meta-analysis of acupuncture versus steroid block therapy favored acupuncture (two studies, n = 144; risk ratio, 1.28; 95% CI, 1.08 to 1.52; p = .005; heterogeneity, I^2^ = 10%) in terms of responder rate.

## Conclusion

Our systematic review and meta-analysis demonstrate that the evidence for acupuncture as a symptomatic therapy of CTS is encouraging but not convincing. The total number of included RCTs and their methodological quality were low. Further rigorous studies are required to establish whether acupuncture has therapeutic value for this indication.

